# Efficacy and safety of traditional Chinese medicine herbal hand-foot bath combined with arnebia oil for chemotherapy-induced hand-foot syndrome refractory to standard treatment: a single-arm prospective interventional pilot study of 30 cases

**DOI:** 10.3389/fmed.2026.1900383

**Published:** 2026-08-21

**Authors:** Ying Chen, Yuqi Jin, Linglin Fu, Ping Song, Ziyan Shao, Yinuo Tan

**Affiliations:** 1 Department of Nursing, The Second Affiliated Hospital, Zhejiang University School of Medicine, Hangzhou, Zhejiang, China; 2 Key Laboratory of Cancer Prevention and Intervention, Department of Medical Oncology, Ministry of Education, The Second Affiliated Hospital, Zhejiang University School of Medicine, Hangzhou, Zhejiang, China; 3 Zhejiang Provincial Clinical Research Center for CANCER, Hangzhou, China; 4 Cancer Center of Zhejiang University, Hangzhou, China; 5 Center for Medical Research and Innovation in Digestive System Tumors, Ministry of Education, Hangzhou, China; 6 Department of Infectious Diseases, The Second Affiliated Hospital, Zhejiang University School of Medicine, Hangzhou, China

**Keywords:** arnebia oil, capecitabine, hand-foot syndrome, pilot study, traditional Chinese medicine herbal bath

## Abstract

**Background:**

Chemotherapy-induced hand-foot syndrome (HFS) is a common dose-limiting cutaneous adverse reaction associated with cytotoxic agents such as capecitabine and small-molecule targeted drugs. Severe HFS may lead to dose reduction or interruption of antitumor treatment, significantly impairing patients' quality of life and treatment compliance. Current standard treatments (urea ointment, vitamin B6, topical corticosteroids, etc.) show limited efficacy for some patients with moderate-to-severe HFS, creating an urgent clinical need for additional supportive interventions.

**Methods:**

This was a single-arm prospective interventional pilot study. It enrolled 30 consecutive patients with solid tumors who received capecitabine-based antitumor therapy or oral targeted therapy, developed HFS ≥ grade 1, and were refractory to standard treatment between November 1, 2025 and April 2026 at the Department of Medical Oncology, The Second Affiliated Hospital of Zhejiang University School of Medicine. All patients received a prespecified combined regimen consisting of self-formulated Yiqi Huoxue Tongluo herbal bath and external arnebia oil. Source-record review showed that the external treatment was initiated during the antitumor drug administration period; for patients receiving capecitabine, treatment generally began on cycle day 2–3 rather than during a scheduled rest period. The primary endpoint was the observed response rate, defined as a reduction in HFS grade by ≥1 grade. Secondary endpoints included complete remission rate, time to improvement, proportion of patients maintaining the original capecitabine dose, two-cycle follow-up recurrence/worsening, and safety. Missing efficacy data were described without imputation.

**Results:**

Thirty patients were enrolled, with a mean age of 59.0±12.4 years and 60.0% male. Baseline HFS grade was grade 1 in 2 patients (6.7%), grade 2 in 24 (80.0%), and grade 3 in 4 (13.3%). Twenty-eight patients had complete efficacy evaluation. The observed overall response rate was 85.7% (24/28), complete remission rate was 7.1% (2/28), and the mean reduction in HFS grade was 0.9. The median time to improvement was 7 days (range 3–28 days). In total, 82.1% (23/28) of evaluable patients maintained the original antitumor regimen without capecitabine dose reduction. During two subsequent treatment cycles of follow-up, no recurrence or worsening of HFS was documented; four patients discontinued capecitabine because of disease change, and 25 patients continued the original capecitabine dose. No severe adverse events related to the combined external regimen were observed.

**Conclusions:**

Yiqi Huoxue Tongluo herbal bath combined with external arnebia oil showed preliminary signals of effectiveness and favorable safety in chemotherapy-induced HFS refractory to standard treatment. Because this single-arm pilot study lacked a concurrent control group, causal inference is limited and the findings should be interpreted as hypothesis-generating pending confirmation in controlled prospective trials.

## 1 Introduction

Hand-foot syndrome (HFS), also known as palmar-plantar erythrodysesthesia, is a frequent dose-limiting cutaneous adverse reaction induced by cytotoxic drugs, such as capecitabine, fluorouracil, and liposomal doxorubicin, as well as small-molecule tyrosine kinase inhibitors. Its incidence varies substantially according to the causative agent, dose, and treatment schedule (1). Typical clinical manifestations include symmetrical erythema, numbness, tingling, desquamation, blistering, and, in severe cases, ulceration of the hands and feet. Severe HFS may impair walking and daily activities and may require dose reduction or interruption of anticancer treatment, thereby affecting quality of life and treatment adherence (2).

Current management of HFS is largely supportive and may include topical moisturizers or urea-based creams, topical corticosteroids, analgesic treatment, and modification of the causative anticancer therapy when clinically necessary. However, evidence supporting many preventive and therapeutic interventions remains limited, and no universally accepted treatment has been established for HFS refractory to standard care (3, 4). Integrative herbal medicine has been investigated as an adjunctive approach for chemotherapy-induced HFS, although the strength of the available evidence remains limited (5). A randomized clinical trial of topical Shouzu Ning Decoction also reported clinical improvement in patients with grade 2 hand-foot skin reaction, supporting further prospective evaluation of external TCM interventions (6).

Based on the consensus-based TCM interpretation of antitumor drug-related HFS, including the pathogenesis theory of “deficiency in origin and excess in superficiality, intermingled stasis and toxin,” our team developed a combined external regimen consisting of self-formulated Yiqi Huoxue Tongluo herbal bath and arnebia oil for patients with HFS refractory to standard treatment (7). This study aimed to preliminarily evaluate its clinical response and safety, providing a basis for future controlled evaluation of this combined regimen.

## 2 Materials and methods

### 2.1 Study design and subjects

This was a single-center, single-arm prospective interventional pilot study. Patients were recruited from inpatients and outpatients of the Department of Medical Oncology, The Second Affiliated Hospital of Zhejiang University School of Medicine between November 1, 2025 and April 2026. Consecutive eligible patients were screened to reduce selection bias. Because this was an exploratory study without a control group, no formal sample-size calculation was performed.

Inclusion Criteria:

1. Pathologically confirmed malignant solid tumor.

2. HFS occurred during capecitabine-containing chemotherapy or targeted therapy.

3. HFS grade ≥1 (per CTCAE 5.0).

4. Refractory to standard treatment, defined as persistent HFS symptoms after topical standard care. In routine practice, standard care consisted of warm-water soaking of the hands and feet followed by topical urea cream; oral celecoxib was added for patients with prominent pain. In the available source records, prior standard-treatment duration was documented for 29 patients and ranged from 7 to 200 days, with a median of 50 days.

5. Age ≥ 18 years and expected survival ≥ 3 months.

6. Karnofsky Performance Status (KPS) score ≥ 50.

7. Signed informed consent and voluntary acceptance of TCM herbal bath.

Exclusion Criteria:

1. Uncontrolled infection or ulceration of hand-foot skin.

2. Severe cardiac, hepatic, or renal insufficiency precluding external bath treatment.

3. Hypersensitivity to any component of the TCM prescription.

4. Pregnancy or lactation.

5. Mental disorders precluding treatment compliance.

### 2.2 Evaluation criteria

1. HFS grading: National Cancer Institute Common Terminology Criteria for Adverse Events (CTCAE), version 5.0 (9).

- Grade 1 (mild): Painless minor skin changes (erythema, edema, hyperkeratosis) or numbness/tingling without interference with daily activities.

- Grade 2 (moderate): Painful skin changes (desquamation, blistering, bleeding, edema) affecting fine motor activities.

- Grade 3 (severe): Severe skin changes (wet desquamation, ulceration, blistering) with intense pain and loss of self-care ability.

2. KPS score: 0–100; higher scores indicate better performance status.

3. Efficacy assessment

- Complete remission (CR): HFS grade reduced to 0 with complete symptom resolution.

- Response: Reduction in HFS grade by ≥1 grade from baseline.

- Non-response: No reduction or worsening of HFS grade.

- Time to improvement: Interval from initiation of the combined external regimen to first subjective symptom improvement.

### 2.3 Intervention

All enrolled patients received a combined external regimen consisting of Yiqi Huoxue Tongluo herbal bath and external arnebia oil.

1. Herbal formula: Astragalus membranaceus 12 g, Angelica sinensis 10 g, Cinnamomum cassia 9 g, Caulis Spatholobi 9 g, Salvia miltiorrhiza 10 g, Carthamus tinctorius 3 g, Paeoniae Radix Rubra 9 g, Dictamnus dasycarpus 6 g, Corydalis decumbens 3 g, and Lycopodiastrum casuarinoides 10 g (prepared as TCM formula granules).

2. Administration: Dissolve granules in 1,500–2,000 mL of warm water at 38–40°C. Soak hands and feet for 15–20 min, twice daily. A 14-day period constituted one treatment course. After soaking, dry hands and feet and apply arnebia oil externally, twice daily. Arnebia oil was a fixed component of the combined regimen for all patients.

3. Antitumor dose adjustment and chemotherapy-cycle timing: The original capecitabine dose was maintained during treatment unless HFS progressed to grade ≥ 3 without relief after symptomatic management, in which case dose reduction or temporary interruption was considered. Source-record review showed that the combined external regimen was initiated during the antitumor drug administration period. For patients receiving capecitabine, external treatment generally began on cycle day 2–3 after approximately four capecitabine doses, and no patient initiated the external treatment during a scheduled capecitabine rest period. Therefore, treatment initiation did not coincide with a planned capecitabine rest period, although chemotherapy-cycle effects could not be fully controlled in this single-arm study.

### 2.4 TCM theory and formulation rationale

In TCM, HFS is categorized as “drug toxin” or “blood bi syndrome.” According to expert consensus on antitumor drug-related HFS, the core pathogenesis can be interpreted as deficiency in origin and excess in superficiality with toxin-induced stasis (7): root deficiency refers to qi-blood insufficiency leading to malnutrition of the distal extremities; superficial excess involves accumulation of drug toxin, intermingled stasis-toxin, and damp-heat fumigating the skin. The therapeutic principle is to replenish qi and activate blood to consolidate the root, and unblock collaterals and resolve toxin to treat the manifestations.

- Monarch drugs: Astragalus membranaceus and Angelica sinensis replenish qi and nourish blood to promote blood circulation. Pharmacological evidence also suggests that Astragalus polysaccharides may exert protective effects on vascular endothelial function (8).

- Minister drugs: Cinnamomum cassia, Caulis Spatholobi, Salvia miltiorrhiza, and Carthamus tinctorius warm and unblock meridians, activate blood and resolve stasis, improving local microcirculation.

- Assistant drugs: Paeoniae Radix Rubra and Dictamnus dasycarpus clear heat and cool blood, dry dampness and resolve toxin, alleviating local erythema and exudation.

- Guide drugs: Corydalis decumbens and Lycopodiastrum casuarinoides unblock collaterals and relax tendons, directing medicinals to the affected extremities.

Combined with external arnebia oil, the regimen may provide additional local wound-healing support. Experimental evidence indicates that shikonin can promote fibroblast and endothelial cell proliferation, angiogenesis, and skin wound healing (10). Because all patients received both components, the present study evaluates the combined regimen and cannot distinguish the independent contribution of the herbal bath from that of arnebia oil.

### 2.5 Statistical analysis

Statistical analysis was performed using SPSS 26.0. Measurement data were expressed as mean ± standard deviation (x̄ ± s) or median (range), depending on distribution. Enumeration data were presented as cases (percentage). Descriptive statistics were used for efficacy and safety analyses. Patients who withdrew before completing efficacy assessment were described in the participant flow and were not included in the primary efficacy analysis. No imputation was performed for missing efficacy data. Given the small sample size and sparse subgroup cell counts, subgroup results were summarized descriptively without formal hypothesis testing.

## 3 Results

### 3.1 Baseline characteristics

**Figure 1:**
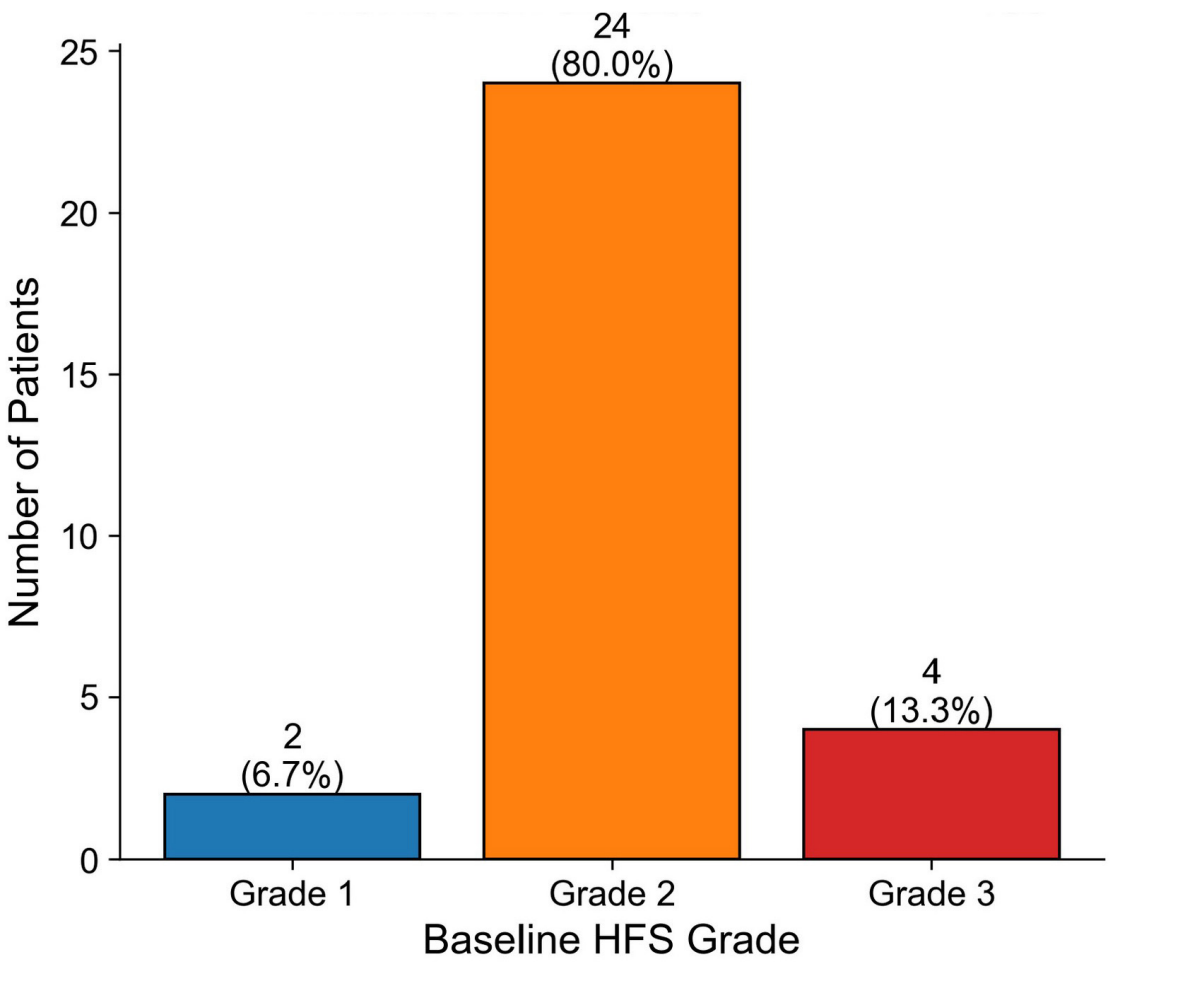
Distribution of baseline HFS grades.

Thirty eligible patients were enrolled. Baseline characteristics are summarized in Table 1. The distribution of baseline HFS grades is shown in Figure 1. Prior standard-treatment duration was available for 29 patients, with a median of 50 days (range 7–200 days; mean 74.4 days); available individual durations are provided in Supplementary Table S1.

**Table 1:** Baseline characteristics of the enrolled patients.

Characteristic	Value (*n* = 30)	Percentage (%)
Age, years (mean ± SD)	59.0 ± 12.4	—
Age range, years	31–84	—
Sex		
Male	18	60.0
Female	12	40.0
Tumor type		
Colorectal cancer	18	60.0
Gastric cancer	3	10.0
Breast cancer	1	3.3
Cholangiocarcinoma	1	3.3
Other solid tumors	7	23.3
Baseline HFS grade		
Grade 1	2	6.7
Grade 2	24	80.0
Grade 3	4	13.3
KPS score (mean ± SD)	71.7 ± 12.1	—
KPS score range	50–90	—
Prior standard-treatment duration, days [median (range); *n* = 29]	50 [7–200]	—

### 3.2 Efficacy outcomes

**Figure 2:**
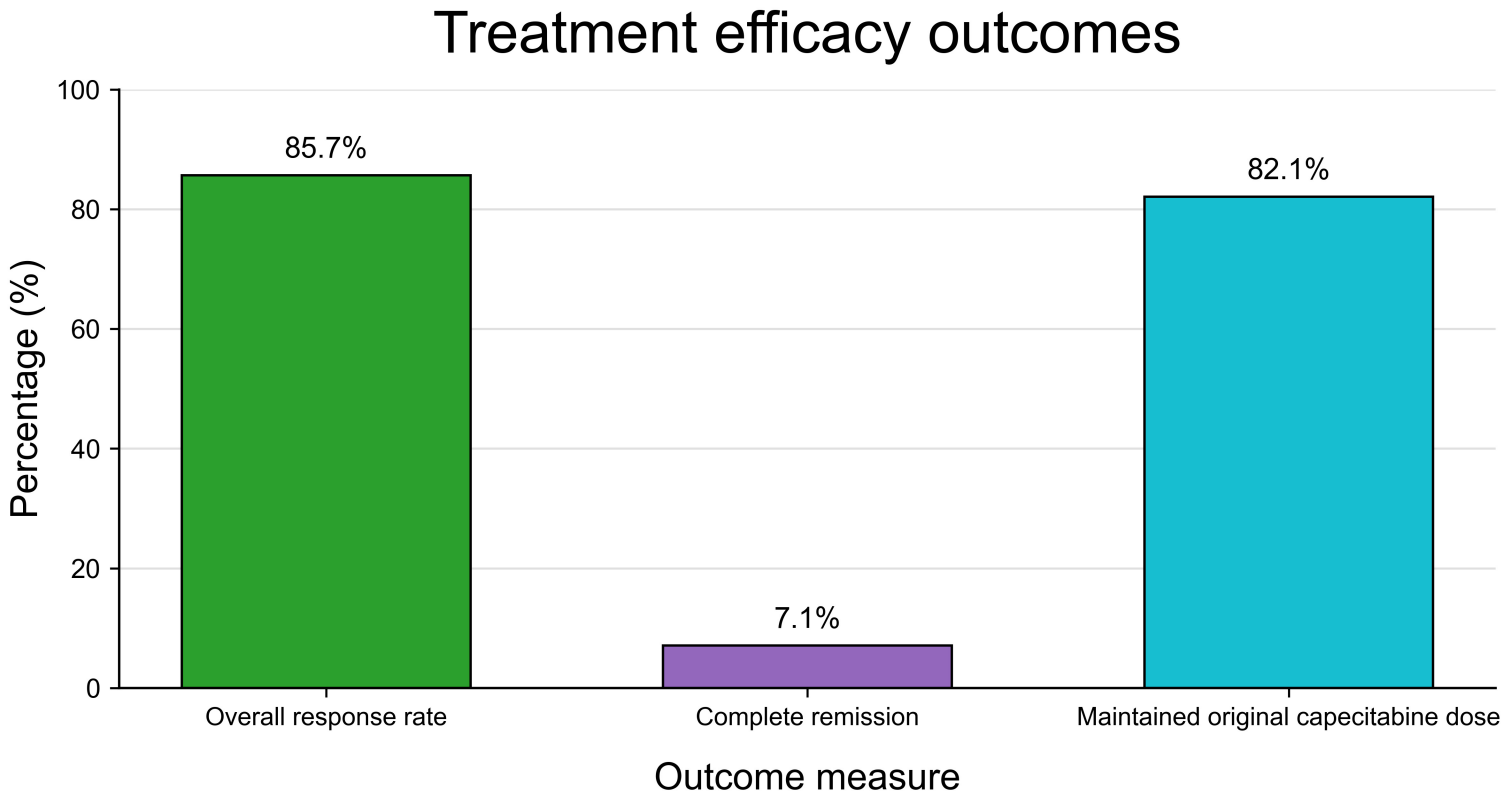
Observed treatment outcomes.

**Figure 3:**
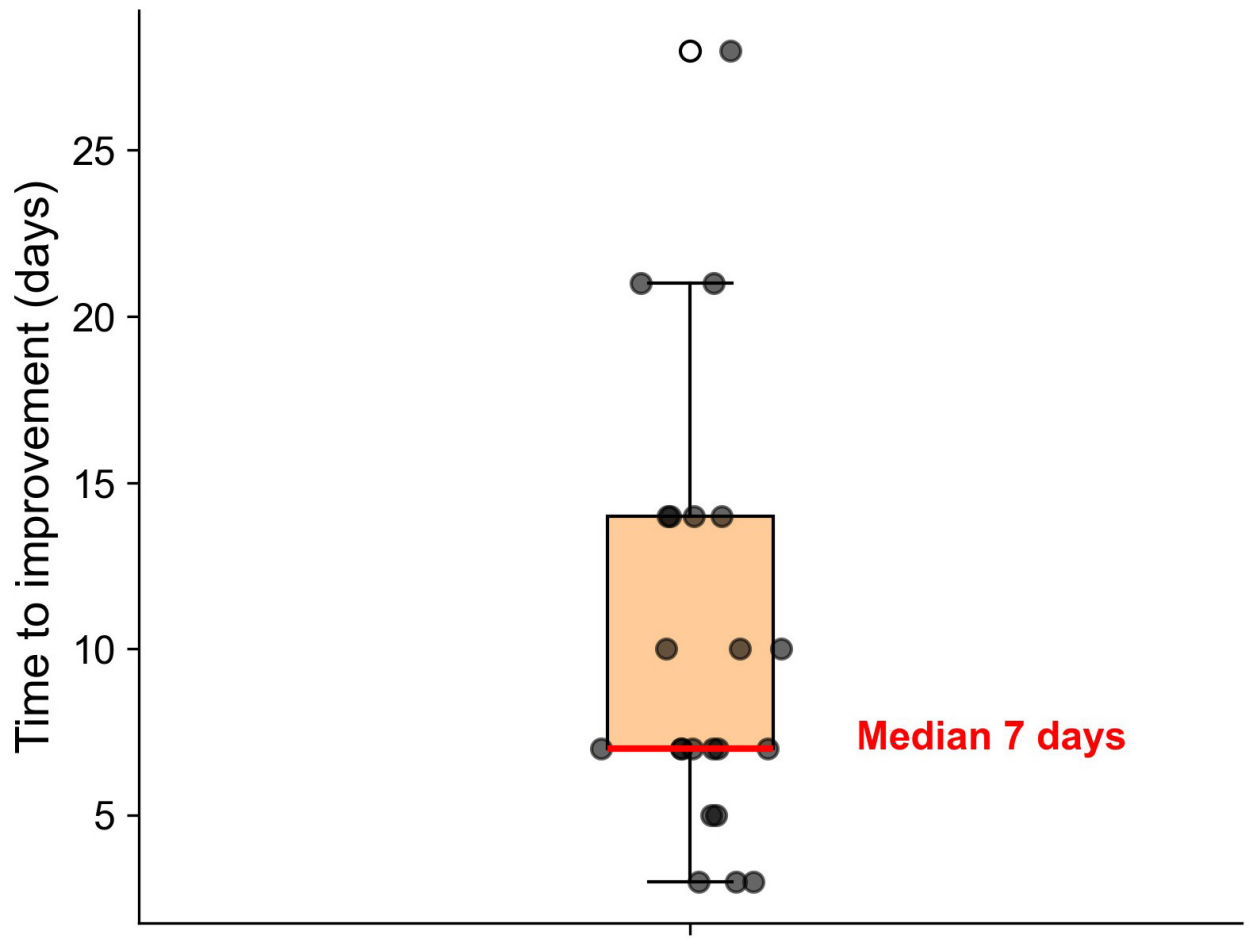
Distribution of time to symptom improvement.

Two patients withdrew early due to disease progression. Twenty-eight patients completed at least one treatment course with complete efficacy data (Table 2). The observed treatment outcomes are shown in Figure 2, and the distribution of time to symptom improvement is presented in Figure 3.

**Table 2:** Efficacy outcomes among evaluable patients.

Efficacy outcome	Value (n=28)	Percentage (%)
Responders (HFS grade reduction ≥1)	24	85.7
Complete remission	2	7.1
Partial response (HFS grade reduction of 1)	22	78.6
Non-response	4	14.3
Mean reduction in HFS grade	0.9	—
Median time to symptom improvement, days	7	—
Time-to-improvement range, days	3–28	—
Maintained original capecitabine dose	23	82.1
Capecitabine dose reduction	5	17.9

### 3.3 Subgroup analysis

**Figure 4:**
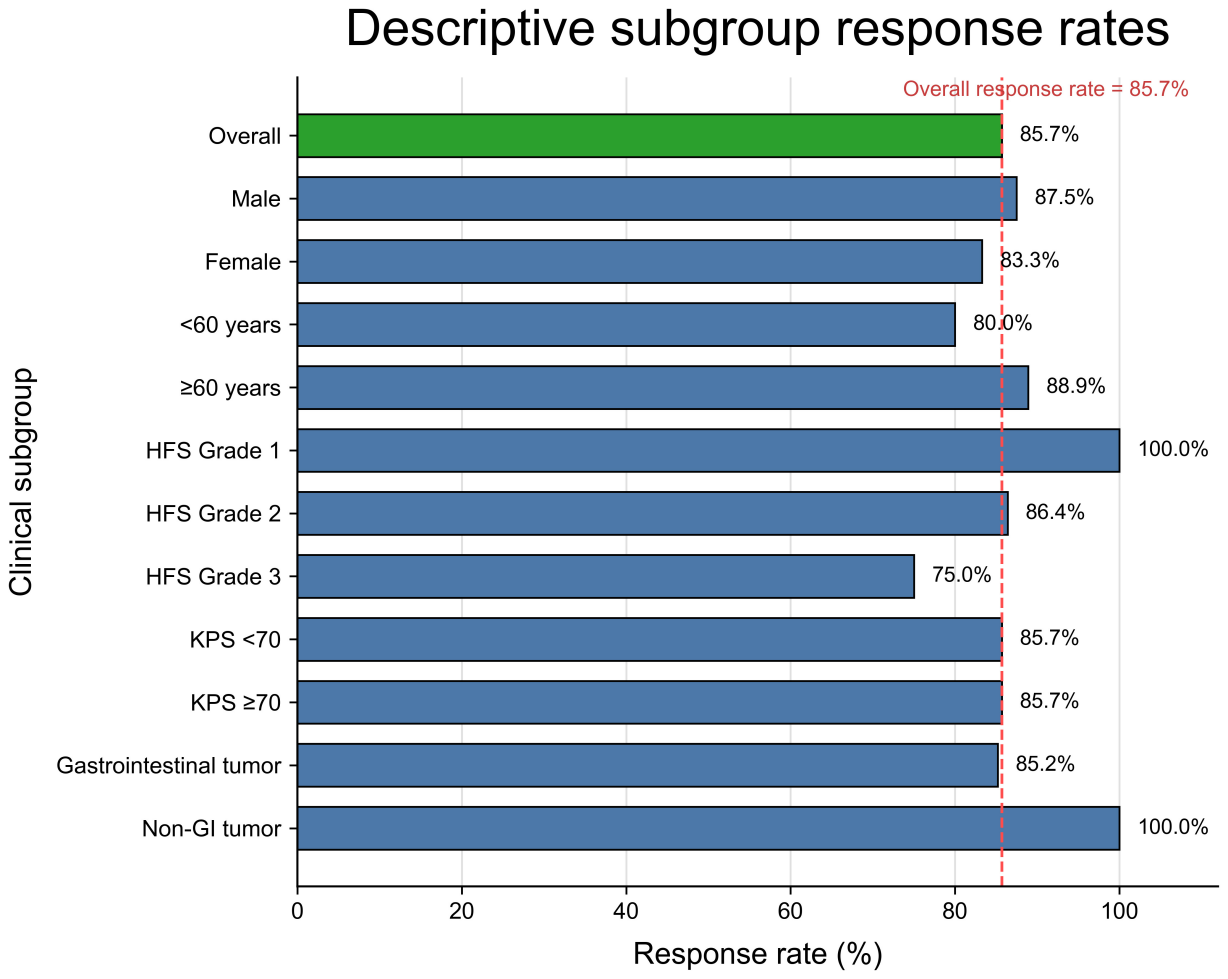
Descriptive subgroup response rates.

- Descriptive efficacy by baseline HFS grade: All 2 patients with baseline grade 1 HFS responded (100.0%); 19 of 22 patients with grade 2 (86.4%) responded; and 3 of 4 patients with grade 3 (75.0%) responded. These subgroup findings are descriptive only because the grade 1 and grade 3 subgroups were very small.

- Descriptive efficacy by tumor type: 23 of 27 patients with gastrointestinal tumors (85.2%) and 1 of 1 patient with a non-gastrointestinal tumor (100.0%) responded.

- Descriptive efficacy by age: 8 of 10 patients aged < 60 years (80.0%) and 16 of 18 patients aged ≥60 years (88.9%) responded.

- Descriptive efficacy by sex: 14 of 16 male patients (87.5%) and 10 of 12 female patients (83.3%) responded.

- Descriptive efficacy by KPS score: 6 of 7 patients with KPS < 70 (85.7%) and 18 of 21 patients with KPS ≥70 (85.7%) responded. No inferential statistical conclusions were drawn from these small subgroups. The descriptive subgroup response rates are shown in Figure 4.

### 3.4 Safety

**Figure 5:**
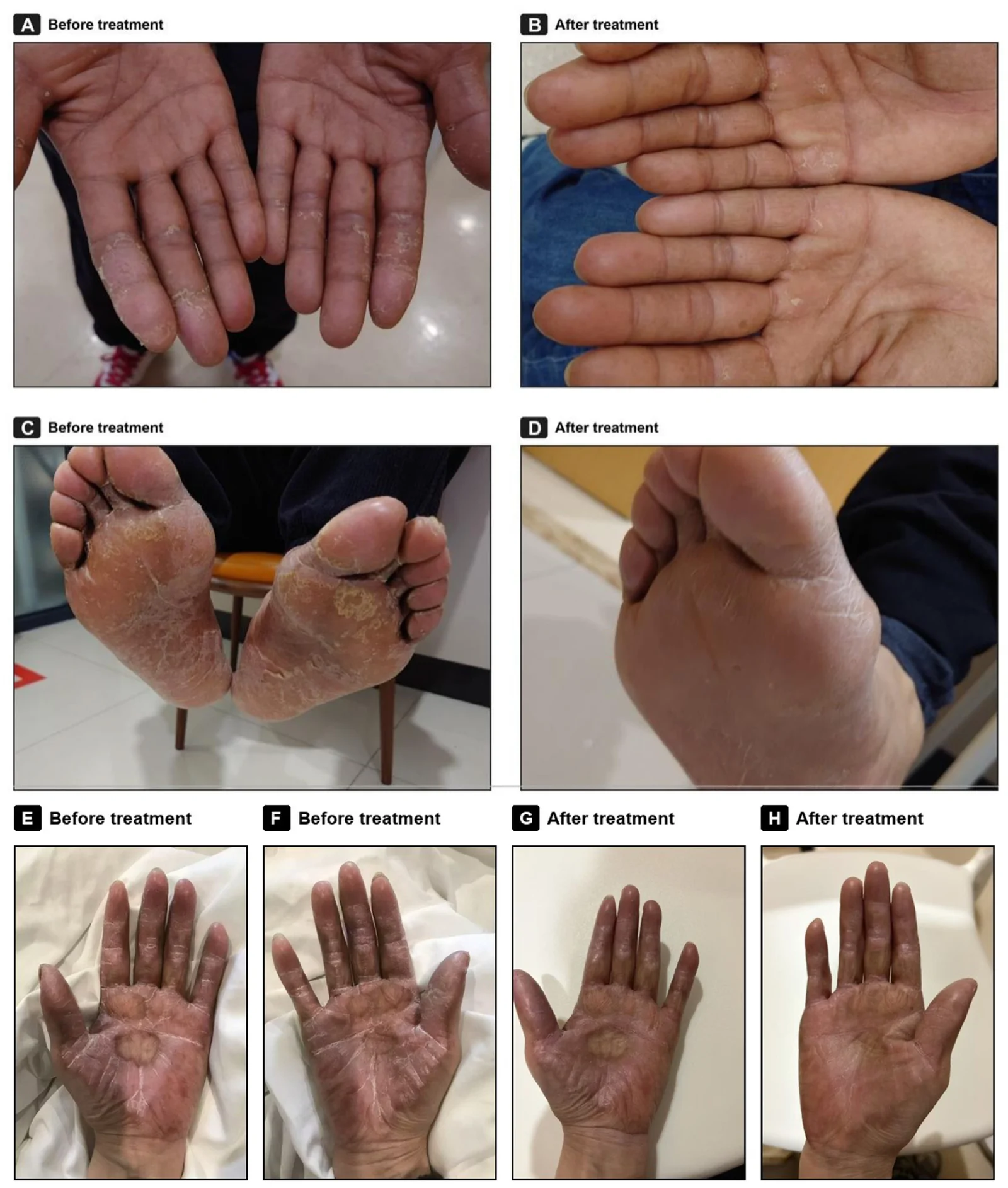
Representative cases before and after treatment. Panels **(A–D)**: representative case of a 59-year-old man with colorectal cancer and chemotherapy-induced HFS grade 2; complete remission was achieved after 2 courses of TCM herbal bath. Panels **(E–H)**: bilateral palmar changes in a second representative case before **(E, F)** and after **(G, H)** treatment.

No severe adverse events related to the combined external regimen were reported during treatment. One patient developed mild local erythema, which resolved after 1 day of temporary interruption without affecting subsequent treatment. One patient reported hand-foot joint pain after soaking and discontinued the herbal bath component; no severe sequelae were documented. Representative clinical changes before and after treatment are shown in Figure 5.

## 4 Discussion

This single-arm prospective interventional pilot study preliminarily suggests that Yiqi Huoxue Tongluo herbal bath combined with external arnebia oil may be associated with symptom improvement in patients with HFS refractory to standard treatment, with an observed response rate of 85.7% and a median time to symptom improvement of 7 days. In addition, 82.1% of evaluable patients maintained the original capecitabine dose for continued antitumor therapy. During two subsequent treatment cycles of follow-up, no recurrence or worsening of HFS was documented, although four patients discontinued capecitabine because of disease change. These findings support further evaluation of this combined regimen, but they should not be interpreted as definitive evidence of efficacy in the absence of a concurrent control group.

The pathogenesis of HFS remains incompletely understood. Proposed mechanisms include direct cytotoxic injury and local drug or metabolite accumulation in acral skin, with inflammatory processes also likely contributing (11). Standard care is mainly supportive, including topical moisturization, corticosteroids, analgesia, and dose modification when clinically indicated (3, 11). Evidence for many preventive and therapeutic interventions remains limited, and persistent or severe HFS may require dose reduction or treatment interruption (4, 11). In our study, patients had persistent symptoms after standard topical care; available source records showed a median prior standard-treatment duration of 50 days (range 7–200 days; *n* = 29). The observed response rate in this uncontrolled pilot study is encouraging, but it cannot be interpreted as superiority over standard care without a randomized comparator.

Integrative herbal medicine has been explored for chemotherapy-induced HFS, but the overall evidence remains limited (5). A randomized clinical trial reported clinical improvement following topical Shouzu Ning Decoction treatment in patients with grade 2 hand-foot skin reaction (6). A recent case report also described topical Chinese medicine combined with urea ointment for regorafenib-induced hand-foot skin reaction, supporting continued clinical interest in external TCM approaches (12). Experimental evidence also suggests that Astragalus polysaccharide may protect vascular endothelial function, although this evidence was obtained from preclinical models and should not be directly extrapolated to clinical HFS (8). Our Yiqi Huoxue Tongluo formula is based on the consensus-supported TCM pathogenesis of “deficiency in origin and excess in superficiality, intermingled stasis and toxin” (6, 7), addressing both root and branch: monarch drugs replenish qi and nourish blood, minister drugs activate blood and unblock collaterals, and assistant drugs clear heat, dry dampness, and resolve toxin.

The descriptive subgroup results should be interpreted with caution. The grade 1 subgroup included only 2 patients, the grade 3 subgroup included only 4 patients, and the non-gastrointestinal tumor subgroup included only 1 patient. We therefore removed formal subgroup *P*-values and present these results only as exploratory descriptive summaries.

This study has limitations. First, it was a single-center, small-sample, single-arm interventional pilot study without a concurrent control group; therefore, no causal inference can be made and the observed 85.7% response rate may partly reflect natural fluctuation, supportive care, or regression of HFS symptoms. Second, although source-record review indicated that external treatment generally began during the capecitabine administration period rather than a scheduled rest period, chemotherapy-cycle effects cannot be fully controlled without a concurrent comparator. Third, all patients received herbal bath plus arnebia oil, so the independent contribution of each component cannot be separated. Fourth, baseline pain and KPS were recorded clinically, but serial patient-reported outcome measures, standardized quality-of-life scales, functional hand-foot scores, and quantitative image analysis were not prespecified. Fifth, follow-up was limited to two subsequent treatment cycles, and long-term durability, recurrence risk beyond this period, PFS, and OS were not evaluated. Future large-sample, prospectively registered randomized controlled trials should include a standard-care control group, stratify or standardize chemotherapy-cycle timing, separate regimen components where feasible, and incorporate multidimensional patient-reported and objective outcomes.

## 5 Conclusions

The combined regimen of self-formulated Yiqi Huoxue Tongluo herbal bath and external arnebia oil showed preliminary response signals and favorable safety for chemotherapy-induced HFS refractory to standard treatment. The regimen may help relieve symptoms and support tolerance to antitumor therapy, but its efficacy should be confirmed in controlled studies before broad clinical recommendation.

## Data Availability

The original contributions presented in the study are included in the article/Supplementary material, further inquiries can be directed to the corresponding authors.

## Supplementary material

Supplementary Table S1 Individual durations of prior standard treatment among the enrolled patients.
