# Supplementary material for: Efficacy and safety of traditional Chinese medicine herbal hand-foot bath combined with arnebia oil for chemotherapy-induced hand-foot syndrome refractory to standard treatment: a single-arm prospective interventional pilot study of 30 cases
Source: Front Med (Lausanne). 2026 Aug 21;13:1900383. doi: 10.3389/fmed.2026.1900383 (PMC13541429; doi:10.3389/fmed.2026.1900383)
Supplement: Supplementary file 1 [file Table_1.docx]

# Supplementary Table S1. Prior standard-treatment duration

De-identified patient-level duration of standard treatment before initiation of the combined external TCM regimen. Standard care consisted of warm-water soaking followed by topical urea cream; oral celecoxib was added for patients with prominent pain. Duration was available for 29 patients.

| Patient ID | Prior standard-treatment duration (days) |
| --- | --- |
| P01 | 56 |
| P02 | 21 |
| P03 | 7 |
| P04 | 14 |
| P05 | 180 |
| P06 | 7 |
| P07 | 180 |
| P08 | 21 |
| P09 | 63 |
| P10 | 180 |
| P11 | 7 |
| P12 | 180 |
| P13 | 10 |
| P14 | 180 |
| P15 | 120 |
| P16 | 13 |
| P17 | 60 |
| P18 | 120 |
| P19 | 50 |
| P20 | 180 |
| P21 | 21 |
| P22 | 200 |
| P23 | 21 |
| P24 | 21 |
| P25 | 56 |
| P26 | 42 |
| P27 | 7 |
| P28 | 120 |
| P29 | 21 |

Summary: median 50 days; range 7-200 days; mean 74.4 days.
